# Supplementary material for: Protective Factors for LGBTI+ Youth Wellbeing: A Scoping Review Underpinned by Recognition Theory
Source: Int J Environ Res Public Health. 2021 Nov 7;18(21):11682. doi: 10.3390/ijerph182111682 (PMC8583439; doi:10.3390/ijerph182111682)
Supplement: Supplementary file 1 [file ijerph-18-11682-s001.zip › Inclusion and exclusion criteria of study selection.pdf]

Inclusion and exclusion criteria of study selection

Inclusion	Exclusion
• Study includes participants who self-identify as lesbian, gay, bisexual, transgender, intersex, queer, questioning, asexual, nonbinary or related terms	• Heterosexual, cisgender participants only<br>• No demographic measure of sexual orientation, gender identity, non-binary or intersex status<br>• No LGBTI+ identification; includes same-gender attraction and/or behaviour
• Study conducted in a country (or region) with a broadly similar Global Acceptance Index rank	• Study conducted in a country (or region) with a widely disparate Global Acceptance Index rank
• Study with participants aged 10-24 years<br>• Study where young people are specifically targeted<br>• Study whereby the mean age falls within the specified age range	• Study whereby participants are children ≤ 10 years or adults ≥ 24 years<br>• Study whereby the mean age falls outside the specified age range
• Study refers to any measures of resilience<br>OR<br>• Study referring to ecological, psychosocial or cognitive measures that protect wellbeing<br>OR<br>• Study referring to factors that mitigate minority stress	• No reference in study to resilience<br>OR<br>• No reference to any protective factors including: interpersonal, community-based or policy/legislative measures<br>OR<br>• No reference to factors that mitigate minority stress<br>OR<br>• Reference to interventions rather than protective factors
• Published in English	• Published in language other than English
• Peer-reviewed	• Non-peer-reviewed
• Academic journal article or dissertation	• Editorial, opinion, book, book chapter, conference abstract, paper or keynote, report etc.
