# Supplementary material for: Protective Factors for LGBTI+ Youth Wellbeing: A Scoping Review Underpinned by Recognition Theory
Source: Int J Environ Res Public Health. 2021 Nov 7;18(21):11682. doi: 10.3390/ijerph182111682 (PMC8583439; doi:10.3390/ijerph182111682)
Supplement: Supplementary file 1 [file ijerph-18-11682-s001.zip › PubMed FINAL search string 21.06.2020.pdf]

PubMed

FINAL search 21 June 2020

#1	LGBT MeSH terms	52,622	48,562
#2	LGBT Title / Abstract	184,016	171,589
#3	#1 OR #2	210,969	196,163
#4	Youth MeSH terms	2,396,441	2,000,031
#5	Youth Title / Abstract	591,162	553,154
#6	#4 OR #5	2,671,060	2,252,053
#7	Wellbeing MeSH terms	239,210	214,423
#8	Wellbeing Title / Abstract	553,115	512,689
#9	#7 OR #8	617,544	568,314
#10	Protective MeSH terms	114,939	105,431
#11	Protective Title /Abstract	3,381,691	3,146,537
#12	#10 OR #11	3,453,290	3,211,157
#13	#3 AND #6	57,508	
#14	#13 AND #9	4,910	
#15	#14 AND #12	1,836	
	Import Endnote 21 June 2020	1,836	

LGBTI+ MeSH Terms

“Sexual and gender minorities"
"gender identity"
Bisexuality
Homosexuality
"Transgender Persons"
Transsexualism
“Intersex condition”
“Intersex conditions”

LGBTI+ Title/Abstract

(Sexual AND gender AND minorit*)
"sexual AND gender minorities"
sexual-minority
gender-minority
homosexual*
"gender identity"
"gender identities"
bisexual*
"transgender persons"
transsexual*
"SGMY"
"SMY"
“GMY"
"LGBT”
"LGBTQ"
"LGBTI"
Lesbigay
"LGB"
“LGBTQ Youth”
"GLBT"
"GLBTQ"
"GLBTI"
Gaylesbi
"GLB"
“GLBT persons”

lesbian*
gay
pansexual*
greysexual*
asexual*
queer
questioning
"sexual minority"
"sexual minorities"
"non-heterosexual"
"non heterosexual"
"same-sex"
"same sex"
transgender*
"gender queer"
gender-queer*
(gender AND queer)
Genderqueer
(same AND gender)
"same gender"
same-gender
"gender variant"
(gender AND non-conform*)
(gender AND nonconform*)
(gender AND fluid*)
(gender AND flexib*)
(gender AND express*)
(gender AND atypical*)
(gender AND creative*)
(gender AND independen*)
Intersex
“Intersex condition”
“Intersex conditions”
“two spirit"
two-spirit
nonbinary
non-binary
“non binary”
“homosexuality and education”
“gay and lesbian studies”
“bisexuality and education”

Youth MeSH Terms
adolescent
adolescence
young adult
young adults
minors

Youth Title/Abstract
adolescent
adolescence
"young adult"
“young adults”
(young AND adult*)
Minors
“Minors (Legal)”

teen*
youth*
(youth OR "young person" OR "young people")
"high school"
"middle school"
“junior high school”
"secondary school"
"post-primary school"
"emerging adult"
“Young Adulthood”
juvenile

Wellbeing MeSH Terms

quality of life
personal satisfaction
mental health

Wellbeing All Fields

“quality of life”
“personal satisfaction”
“mental health”
wellness
well-being
wellbeing
“Well Being”
“happy with life”
“happiness with life”
“life satisfaction”
(Life AND satisf*)
(quality AND life)

Protective factors MeSH Terms

“psychological resilience”
“social environment”
“social environments”
psychosocial support systems
psychosocial support system

Protective factors All Fields

social environment*
“psychological resilience”
resilien*
resilient
resilience
resiliency
“Resilience (Psychological)”
“Resilience (psychology)”
Hardiness
(psychological AND (endurance OR hardiness OR adapt* OR adjust*)
“Psychological Processes and Principles”
“Protective factors”
(protect* AND factor*)
resourceful*
improv*
assist*
protect
promote
“social support”
“psychosocial support”
“psychosocial support system”

“psychosocial support systems”

FINAL PubMed Search
((((((((((“Sexual and gender minorities"[MeSH Terms]) OR ("gender identity"[MeSH Terms])) OR (Bisexuality[MeSH Terms])) OR (homosexuality[MeSH Terms])) OR ("Transgender Persons"[MeSH Terms])) OR (transsexualism[MeSH Terms])) OR (“Intersex condition”[MeSH Terms])) OR (“Intersex conditions”[MeSH Terms])) OR

((Sexual[Title/Abstract] AND gender[Title/Abstract] AND minorit*)[Title/Abstract]) OR ("sexual[Title/Abstract] AND gender minorities"[Title/Abstract])) OR (sexual-minority[Title/Abstract])) OR (gender-minority[Title/Abstract])) OR (homosexual*[Title/Abstract])) OR ("gender identity"[Title/Abstract])) OR ("gender identities"[Title/Abstract])) OR (bisexual*[Title/Abstract])) OR ("transgender persons"[Title/Abstract])) OR (transsexual*[Title/Abstract])) OR ("SGMY"[Title/Abstract])) OR ("SMY"[Title/Abstract])) OR (“GMY"[Title/Abstract])) OR ("LGBT”[Title/Abstract])) OR ("LGBTQ"[Title/Abstract])) OR ("LGBTI"[Title/Abstract])) OR (Lesbigay[Title/Abstract])) OR ("LGB"[Title/Abstract])) OR (“LGBTQ Youth”[Title/Abstract])) OR ("GLBT"[Title/Abstract])) OR ("GLBTQ"[Title/Abstract])) OR ("GLBTI"[Title/Abstract])) OR (Gaylesbi[Title/Abstract])) OR ("GLB"[Title/Abstract])) OR (“GLBT persons”[Title/Abstract])) OR (lesbian*[Title/Abstract])) OR (gay[Title/Abstract])) OR (pansexual*[Title/Abstract])) OR (greysexual*[Title/Abstract])) OR (asexual*[Title/Abstract])) OR (queer[Title/Abstract])) OR (questioning[Title/Abstract])) OR ("sexual minority"[Title/Abstract])) OR ("sexual minorities"[Title/Abstract])) OR ("non-heterosexual"[Title/Abstract])) OR ("non heterosexual"[Title/Abstract])) OR ("same-sex"[Title/Abstract])) OR ("same sex"[Title/Abstract])) OR (transgender*[Title/Abstract])) OR ("gender queer"[Title/Abstract])) OR (gender-queer*[Title/Abstract])) OR ((gender[Title/Abstract] AND queer)[Title/Abstract])) OR (Genderqueer[Title/Abstract])) OR ((same[Title/Abstract] AND gender)[Title/Abstract])) OR ("same gender"[Title/Abstract])) OR (same-gender[Title/Abstract])) OR ("gender variant"[Title/Abstract])) OR ((gender[Title/Abstract] AND non-conform*)[Title/Abstract])) OR ((gender[Title/Abstract] AND nonconform*)[Title/Abstract])) OR ((gender[Title/Abstract] AND fluid*)[Title/Abstract])) OR ((gender[Title/Abstract] AND flexib*)[Title/Abstract])) OR ((gender[Title/Abstract] AND express*)[Title/Abstract])) OR ((gender[Title/Abstract] AND atypical*)[Title/Abstract])) OR ((gender[Title/Abstract] AND creative*)[Title/Abstract])) OR ((gender[Title/Abstract] AND independen*)[Title/Abstract])) OR (Intersex[Title/Abstract])) OR (“Intersex condition”[Title/Abstract])) OR (“Intersex conditions”[Title/Abstract])) OR (“two spirit"[Title/Abstract])) OR (two-spirit[Title/Abstract])) OR (nonbinary[Title/Abstract])) OR (non-binary[Title/Abstract])) OR (“non binary”[Title/Abstract])) OR (“homosexuality[Title/Abstract] AND education”[Title/Abstract])) OR (“gay[Title/Abstract] AND lesbian studies”[Title/Abstract])) OR (“bisexuality[Title/Abstract] AND education”[Title/Abstract])))

AND

((((((adolescent[MeSH Terms]) OR (adolescence[MeSH Terms])) OR (young adult[MeSH Terms])) OR (young adults[MeSH Terms])) OR (minors[MeSH Terms]))

OR ((((((((((((((((((adolescent[Title/Abstract]) OR (adolescence[Title/Abstract])) OR ("young adult"[Title/Abstract])) OR (“young adults”[Title/Abstract])) OR ((young[Title/Abstract] AND adult*)[Title/Abstract])) OR (Minors[Title/Abstract])) OR (“Minors (Legal)”[Title/Abstract])) OR (teen*[Title/Abstract])) OR (youth*[Title/Abstract])) OR ((youth[Title/Abstract] OR "young person"[Title/Abstract] OR "young people")[Title/Abstract])) OR ("high school"[Title/Abstract])) OR ("middle school"[Title/Abstract])) OR (“junior high school”[Title/Abstract])) OR ("secondary school"[Title/Abstract])) OR ("post-primary school"[Title/Abstract])) OR ("emerging adult"[Title/Abstract])) OR (“Young Adulthood”[Title/Abstract])) OR (juvenile[Title/Abstract]))))

AND

(((((quality of life[MeSH Terms]) OR (personal satisfaction[MeSH Terms])) OR (mental health[MeSH Terms]))

OR ((((((((((((“quality of life”[Title/Abstract]) OR (“personal satisfaction”[Title/Abstract])) OR (“mental health”[Title/Abstract])) OR (wellness[Title/Abstract])) OR (well-being[Title/Abstract])) OR (wellbeing[Title/Abstract])) OR (“Well Being”[Title/Abstract])) OR (“happy with life”[Title/Abstract])) OR (“happiness with life”[Title/Abstract])) OR (“life satisfaction”[Title/Abstract])) OR ((Life[Title/Abstract] AND satisf*)[Title/Abstract])) OR ((quality[Title/Abstract] AND life)[Title/Abstract])))

AND

((((((“psychological resilience”[MeSH Terms]) OR (“social environment”[MeSH Terms])) OR (“social environments”[MeSH Terms])) OR (psychosocial support systems[MeSH Terms])) OR (psychosocial support system[MeSH Terms]))

OR ((((((((((((((((((((((("social environment*"[Title/Abstract]) OR (“psychological resilience”[Title/Abstract])) OR (resilien*[Title/Abstract])) OR (resilient[Title/Abstract])) OR (resilience[Title/Abstract])) OR (resiliency[Title/Abstract])) OR (“Resilience (Psychological)”[Title/Abstract])) OR (“Resilience (psychology)”[Title/Abstract])) OR (Hardiness[Title/Abstract])) OR ((psychological[Title/Abstract] AND (endurance[Title/Abstract] OR hardiness[Title/Abstract] OR adapt*[Title/Abstract] OR

adjust*)[Title/Abstract])) OR (“Psychological Processes[Title/Abstract] AND Principles”[Title/Abstract])) OR (“Protective factors”[Title/Abstract])) OR ((protect*[Title/Abstract] AND factor*)[Title/Abstract])) OR (resourceful*[Title/Abstract])) OR (improv*[Title/Abstract])) OR (assist*[Title/Abstract])) OR (protect[Title/Abstract])) OR (promote[Title/Abstract])) OR (“social support”[Title/Abstract])) OR (“psychosocial support”[Title/Abstract])) OR (“psychosocial support system”[Title/Abstract])) OR (“psychosocial support systems”[Title/Abstract])))) Filters: English
