# Supplementary material for: Protective Factors for LGBTI+ Youth Wellbeing: A Scoping Review Underpinned by Recognition Theory
Source: Int J Environ Res Public Health. 2021 Nov 7;18(21):11682. doi: 10.3390/ijerph182111682 (PMC8583439; doi:10.3390/ijerph182111682)
Supplement: Supplementary file 1 [file ijerph-18-11682-s001.zip › Screening template using PCC criteria and inclusion criteria.pdf]

Screening template using PCC criteria and inclusion criteria

Document review

Reviewer:

Article title:____

Study population: LGBTI+ identity

☐ Sexual minority youth (sexual orientation)

☐ Gender minority youth (gender identity)

☐ Variations of spectrum of sex development (intersex)

☐ Unsure

☐ None of the above (exclude: not population)

Notes to explain decision (optional):

____

Study population: age

☐ Participants are aged between 10-24 years

☐ Targets young people

☐ The mean age of study participants is between the specified age range

☐ Unsure

☐ None of the above (exclude: not population)

Notes to explain decision (optional):

____

Study concept: wellbeing (including protective factors) OR mental health (including resilience and factors that mitigate minority stress - see diagram)

☐ Yes

☐ Unsure

☐ No (exclude: not concept)

Notes to explain decision (optional):

____

Study context: Global Acceptance Index rank 6.8-8.9 – see chart

☐ Yes

☐ Unsure

☐ No (exclude – not context)

Notes to explain decision (optional):

____

Note: If not population, not concept, not context – exclude – not relevant)

Study language: published in English?

☐ Yes

☐ Unsure

☐ No (exclude – not language)

Notes to explain decision (optional):

____

Study peer review: published in an academic journal or peer reviewed as part of PhD defence?

☐ Yes

☐ Unsure

☐ No (exclude – not peer reviewed)

Notes to explain decision (optional):

____
