# Supplementary figures and images for: Protective Factors for LGBTI+ Youth Wellbeing: A Scoping Review Underpinned by Recognition Theory
Source: Int J Environ Res Public Health. 2021 Nov 7;18(21):11682. doi: 10.3390/ijerph182111682 (PMC8583439; doi:10.3390/ijerph182111682)

# Overview of inclusion and exclusion criteria for title and abstract screening

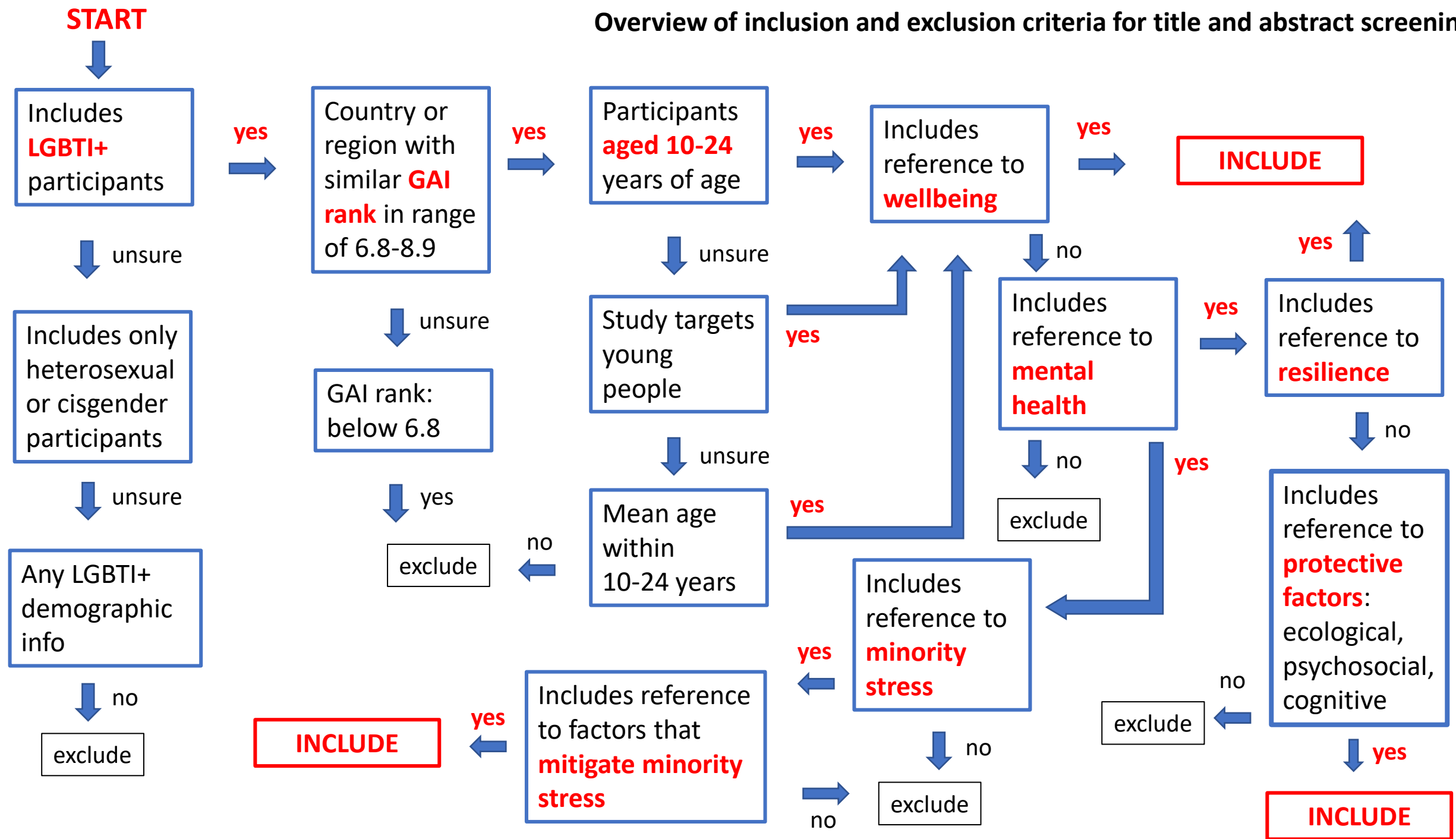

Supplement: Supplementary file 1 [file ijerph-18-11682-s001.zip › Flow chart overview of inclusion and exclusion criteria for title and abstract screening.pdf]
